# Clustering single-cell multi-omics data via graph regularized multi-view ensemble learning

**DOI:** 10.1093/bioinformatics/btae169

**Published:** 2024-03-28

**Authors:** Fuqun Chen, Guanhua Zou, Yongxian Wu, Le Ou-Yang

**Affiliations:** College of Electronic and Information Engineering, Shenzhen University, Shenzhen 518060, Guangdong, China; Guangdong Key Laboratory of Intelligent Information Processing, Shenzhen University, Shenzhen 518060, Guangdong, China; Shenzhen Key Laboratory of Media Security and Guangdong Laboratory of Artificial Intelligence and Digital Economy (SZ), Shenzhen University, Shenzhen 518060, Guangdong, China; College of Electronic and Information Engineering, Shenzhen University, Shenzhen 518060, Guangdong, China; Guangdong Key Laboratory of Intelligent Information Processing, Shenzhen University, Shenzhen 518060, Guangdong, China; Shenzhen Key Laboratory of Media Security and Guangdong Laboratory of Artificial Intelligence and Digital Economy (SZ), Shenzhen University, Shenzhen 518060, Guangdong, China; College of Electronic and Information Engineering, Shenzhen University, Shenzhen 518060, Guangdong, China; Guangdong Key Laboratory of Intelligent Information Processing, Shenzhen University, Shenzhen 518060, Guangdong, China; Shenzhen Key Laboratory of Media Security and Guangdong Laboratory of Artificial Intelligence and Digital Economy (SZ), Shenzhen University, Shenzhen 518060, Guangdong, China; College of Electronic and Information Engineering, Shenzhen University, Shenzhen 518060, Guangdong, China; Guangdong Key Laboratory of Intelligent Information Processing, Shenzhen University, Shenzhen 518060, Guangdong, China; Shenzhen Key Laboratory of Media Security and Guangdong Laboratory of Artificial Intelligence and Digital Economy (SZ), Shenzhen University, Shenzhen 518060, Guangdong, China

## Abstract

**Motivation:**

Single-cell clustering plays a crucial role in distinguishing between cell types, facilitating the analysis of cell heterogeneity mechanisms. While many existing clustering methods rely solely on gene expression data obtained from single-cell RNA sequencing techniques to identify cell clusters, the information contained in mono-omic data is often limited, leading to suboptimal clustering performance. The emergence of single-cell multi-omics sequencing technologies enables the integration of multiple omics data for identifying cell clusters, but how to integrate different omics data effectively remains challenging. In addition, designing a clustering method that performs well across various types of multi-omics data poses a persistent challenge due to the data’s inherent characteristics.

**Results:**

In this paper, we propose a graph-regularized multi-view ensemble clustering (GRMEC-SC) model for single-cell clustering. Our proposed approach can adaptively integrate multiple omics data and leverage insights from multiple base clustering results. We extensively evaluate our method on five multi-omics datasets through a series of rigorous experiments. The results of these experiments demonstrate that our GRMEC-SC model achieves competitive performance across diverse multi-omics datasets with varying characteristics.

**Availability and implementation:**

Implementation of GRMEC-SC, along with examples, can be found on the GitHub repository: https://github.com/polarisChen/GRMEC-SC.

## 1 Introduction

Cells serve as the fundamental units of living organisms, where metabolic activities and genetic reproduction take place (Zeng and Sanes 2017). Understanding cell type information is crucial for comprehending cellular mechanisms and growth trajectories (Kiselev *et al.* 2019). Moreover, cell type information is often required for various downstream analyses, including developmental trajectory analysis and cancer subtype recognition (Kiselev *et al.* 2019). However, determining cell types is often challenging as they are usually unknown in advance. Gene expression profiling provides an opportunity to accurately distinguish between cell types (Lacey *et al.* 1992, Trapnell *et al.* 2014), but traditional bulk sequencing technologies cannot capture and analyze individual cells effectively (Brawand *et al.* 2011). The emergence of single-cell sequencing makes it feasible to study transcriptome heterogeneity among cells through single-cell RNA sequencing (scRNA-seq) (Jaitin *et al.* 2014), paving the way for identifying cell types by clustering scRNA-seq data (Stegle *et al.* 2015, Kiselev *et al.* 2017).

Clustering scRNA-seq data faces a number of challenges (Kiselev *et al.* 2019), such as high dimensionality (Bellman 1966), dropout events (Andrews and Hemberg 2019), and high noise rate. Recently, numerous computational approaches have been developed for clustering scRNA-seq data. For instance, CountClust (Dey *et al.* 2017) clusters cells by referring to a generative probabilistic model, i.e. latent Dirichlet allocation. CIDR (Lin *et al.* 2017) calculates correlated distances and performs hierarchical clustering on scRNA-seq data against dropout events. Seurat (Stuart *et al.* 2019) improves clustering accuracy by considering k-nearest-neighbors (k-NN) (Altman 1992) of spatially resolved gene expression profiling. SIMLR (Wang *et al.* 2017) focuses on learning a distance map with multi-kernel learning.

The above methods solely rely on transcriptome information (i.e. scRNA-seq data) to distinguish cell types, but the quality of scRNA-seq data limits their performance. Recently, the advent of single-cell multi-omics sequencing technologies, such as SHARE-seq (Ma *et al.* 2020), SNARE-seq (Chen *et al.* 2019), and CITE-seq (Stoeckius *et al.* 2017), enables the measurement of multiple omics (e.g. gene expression and chromatin accessibility) within individual cells simultaneously. As different omics data may capture the cell type information from different aspects, integrating multiple omics data can lead to more accurate and comprehensive clustering results, and provide deeper insights into cellular heterogeneity (Nie *et al.* 2017a,b, Tao *et al.* 2017, Luo *et al.* 2018, Zou *et al.* 2022, Zhan *et al.* 2023). Various computational approaches have been developed to cluster single-cell multi-omics data. For example, BREM-SC (Wang *et al.* 2020) utilizes a Bayesian random effects mixture model to jointly cluster scRNA-seq and antibody-derived tags (ADTs) data obtained from CITE-seq. DCCA (Zuo *et al.* 2021) combines variational autoencoders (VAEs) and attention-transfer to jointly analyze scRNA-seq and single-cell genomics (scEpigenomics) data from the same cell.

While the aforementioned methods have offered valuable tools for cell clustering, finding a method that performs effectively in all scenarios remains challenging due to their model assumptions and the diversity of data distributions. For example, BREM-SC may deliver satisfactory performance on CITE-seq datasets but exhibit poor performance on scATAC-seq datasets. Consensus clustering, on the other hand, can utilize clustering results obtained from various base clustering methods to improve clustering performance (Strehl and Ghosh 2002). However, existing consensus clustering methods heavily depend on the base clustering results.

To tackle the aforementioned challenges, we introduce a novel Graph Regularized Multi-view Ensemble Clustering (GRMEC-SC) model for clustering single-cell multi-omics data. Initially, we use multiple mono-omic clustering methods on each omic data independently to derive the base clustering results, which serve as guidance for learning the consensus co-cluster affinity matrix. To consider the information inherent in the original omics data, we introduce a weighted ensemble nonnegative matrix factorization (NMF) (Lee and Seung 1999, Nie *et al.* 2017a,b) strategy to extract the consensus low-dimensional representations of cells from multiple omics data. Subsequently, the consensus low-dimensional representations and the consensus co-cluster affinity matrix learned from multi-omics data and multiple base clustering results are integrated into two graph regularization terms to facilitate the learning of the final cluster indicator matrix. To showcase the effectiveness of our GRMEC-SC model, we perform experiments on five real-world multi-omics datasets. The experimental results illustrate that our GRMEC-SC model can deliver consistent and competitive performance across a range of datasets with diverse characteristics, outperforming existing state-of-the-art mono-omic and multi-omics clustering algorithms.

## 2 Materials and method

**Figure 1.**
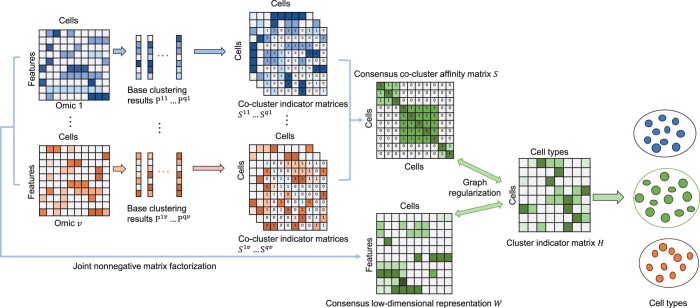
Schematic overview of the proposed GRMEC-SC model. Initially, multiple base clustering methods are applied to each omic data separately to produce base clustering results. Then the consensus low-dimensional representation matrix *W* and the consensus co-cluster affinity matrix *S* are learned from multi-omics data and multiple base clustering results, respectively. Finally, they are incorporated into two graph regularization terms to guide the learning of cluster indicator matrix *H*.

In this section, we will outline the details of our Graph Regularized Multi-view Ensemble Clustering (GRMEC-SC) model. The flowchart of our model is illustrated in Fig. 1. We will commence by formulating the problem, followed by an in-depth explanation of our GRMEC-SC model and the derivation of its optimization scheme. Lastly, we will analyze the overall complexity of the model.

### 2.1 Problem formulation

Throughout this paper, matrices are written in upper-case letters and scalars in lower-case letters. The (*i*, *j*)th entry of a matrix *X* are denoted as $X_{ij}$. The *i*th row or *j*th column of a matrix *X* are expressed as $X_{i\cdot }$ or $X_{\cdot j}$. $X^{T}$ denotes the transpose of matrix *X*. tr(X) stands for the trace of matrix *X*. $||X|{|}_{F}$ represents the Frobenius norm of matrix *X*. Besides, $1_{n}$ denotes a column vector whose elements are all one.

Given a single-cell multi-omics dataset including *m* omics data, each omic data captures the information of $p_{v}$ features for the same set of *n* cells, which can be represented as a matrix $X^{v}\in R^{n\times p_{v}}$, 1 ≤ v ≤ m. As all the entries in $X^{v}$ are nonnegative, we adopt NMF (Lee and Seung 1999, Wang and Zhang 2013) to extract low-dimensional representations of cells from each omic data $X^{v}$. Note that different omics data describe the same set of cells from different aspects. To extract the intrinsic consistent information inherent in multiple omics data to detect cell clusters more accurately, we introduce the following joint NMF (Zhang *et al.* 2012) framework:

(1) $$\begin{array}{c} \begin{array}{cc} \underset{W,\{V^{v}\}}{\min} & \sum_{v}||X^{v}-WV^{v}|{|}_{F}^{2},\\ s.t. & \;W\;>\;0,V^{v}\;>\;0,v=1,\ldots ,m, \end{array} \end{array}$$

where $W\in R_{+}^{n\times k}$ denotes the consensus low-dimensional representations of cells learned from multiple omics data and $V^{v}\in R_{+}^{k\times p_{v}}$ denotes the corresponding basis in the feature space of each omic data, and *k* is the dimension of latent representations which is set to 50 by default. The impact of this parameter is demonstrated in the Supplementary Materials.

Due to inherent noise and biases in single-cell sequencing technologies, the reliability of various omics data may vary. To prevent the model from being misled by noisy information and to enhance the integration of multiple omics data effectively, we assign different weights to each omics data and introduce the following weighted fusion strategy:

(2) $$\begin{array}{c} \begin{array}{cc} \underset{W,\{V^{v}\},\{w_{v}\}}{\min} & \sum_{v}w_{v}||X^{v}-WV^{v}|{|}_{F}^{2},\\ s.t. & \;W\;>\;0,V^{v}\;>\;0,v=1,\ldots ,m, \end{array} \end{array}$$

where $w_{v}$ is the weight assigned to *v*th omic data. To learn these weights adaptively from the data, following (Nie *et al.* 2017a), we adopt the following function to calculate $w_{v}$:

(3) $$\begin{array}{c} \begin{array}{c} w_{v}=\frac{1}{2||X^{v}-WV^{v}|{|}_{F}}. \end{array} \end{array}$$

To identify cell clusters from *W*, let $H\in R_{+}^{n\times C}$ denote the cluster indicator matrix, where *C* is the number of clusters. By enforcing $\sum_{c=1}^{C}H_{ic}=1$ for i=1,…,n, each entry $H_{ic}$ of *H* denotes the probability that *i*th cell belongs to *c*th cluster. Based on *H*, we can calculate an affinity matrix $S^{H}=HH^{T}$, which captures the co-cluster similarities between cells. The higher the value of $S_{ij}^{H}$, the more likely it is that the *i*th and *j*th cells belong to the same cluster.

To learn *W* and *H* adaptively, by adding a graph regularization term to Equation (2) (Cai *et al.* 2010), we formulate the following objective function:

(4) $$\begin{array}{c} \begin{array}{cc} \underset{W,\{V^{v}\},H,\{w_{v}\}}{\min}L_{MV} & =\sum_{v}w_{v}||X^{v}-WV^{v}|{|}_{F}^{2}\\ & +\frac{1}{2}\lambda_{1}\sum_{i,j}||W_{i.}-W_{j.}|{|}_{F}^{2}S_{ij}^{H}\\ & =\sum_{v}w_{v}||X^{v}-WV^{v}|{|}_{F}^{2}+\lambda_{1}tr(W^{T}L_{H}W),\\ s.t. & W\;>\;0,V^{v}\;>\;0,H\;>\;0,\\ & H_{i\cdot }1_{c}=1,v=1,\ldots ,m, \end{array} \end{array}$$

where $L_{H}=D^{H}-S^{H}$ is the Laplacian matrix of $S^{H}$ and $D^{H}$ is a diagonal matrix with $D_{ii}^{H}=\sum_{j=1}^{n}S_{ij}^{H}$. By minimizing Equation (4), cells with similar representations will have similar cluster assignments. This approach allows the representation learning process and the clustering process to mutually guide each other, leading to more precise results.

As various clustering approaches have been devised to identify cell clusters from mono-omic data like scRNA-seq data, we can generate multiple base clustering results by using different clustering models on a specific type of omic data. To leverage the insights extracted by diverse models, we introduce an ensemble learning strategy to integrate multiple base clustering results into our model. In particular, we apply different base clustering models on each omic data $X^{v}$ to generate the base clustering results. The *r*th base clustering result on *v*th omic data is denoted as $P^{rv},1\;\leq \;r\;\leq \;q,1\;\leq \;v\;\leq \;m$, where $P^{rv}=\{p_{1}^{rv},\ldots ,p_{n}^{rv}\}$ and $p_{i}^{rv}$ denotes the cluster label to which the *i*th cell is assigned. Then the base clustering result is transformed into a co-cluster indicator matrix $S^{rv}\in {\left\{0,1\right\}}^{n\times n}$:

(5) $$\begin{array}{c} S_{ij}^{rv}=\{\begin{array}{cc} 1, & if\;p_{i}^{rv}=p_{j}^{rv},\\ 0, & \mathrm{else}. \end{array} \end{array}$$

The following loss function is utilized to learn a consensus co-cluster affinity matrix *S* from various base clustering results $S^{rv}$:

(6) $$\begin{array}{c} \begin{array}{cc} \underset{S}{\min} & \sum_{r,v}||S-S^{rv}|{|}_{F}^{2},\\ s.t. & \;S\;>\;0. \end{array} \end{array}$$

Here, each entry $S_{ij}$ of *S* denotes the co-cluster propensity between *i*th and *j*th cells learned from multiple base clustering results. If all base clustering models consider these two cells to belong to the same cluster, they will achieve high $S_{ij}$. Note that different base clustering results behave differently on capturing the true cell clusters. Thus, to integrate multiple base clustering results effectively and adaptively, instead of treating each base clustering equally, we introduce the following weighted ensemble learning approach:

(7) $$\begin{array}{c} \begin{array}{cc} \underset{S,\alpha_{rv}}{\min} & \sum_{r,v}\alpha_{rv}||S-S^{rv}|{|}_{F}^{2},\\ s.t. & \;S\;>\;0, \end{array} \end{array}$$

where $\alpha_{rv}$ is defined as:

(8) $$\begin{array}{c} \begin{array}{c} \alpha_{rv}=\frac{1}{2||S-S^{rv}|{|}_{F}}. \end{array} \end{array}$$

The self-weighting approach described above eliminates the need for additional parameters, thereby reducing the complexity of tuning. To utilize *S* to guide the learning of cluster indicator matrix *H*, we impose a graph regularization term on the above loss function and obtain the following objective function:

(9) $$\begin{array}{c} \begin{array}{cc} \underset{S,H,\alpha_{rv}}{\min} & L_{EC}=\sum_{r,v}\alpha_{rv}||S-S^{rv}|{|}_{F}^{2}+\lambda_{2}tr(H^{T}L_{S}H),\\ s.t. & \;S\;>\;0,H\;>\;0,H_{i\cdot }1_{c}=1, \end{array} \end{array}$$

where $L_{S}=D^{S}-S$ is the Laplacian matrix of *S* and $D^{S}$ is a diagonal matrix with $D_{ii}^{S}=\sum_{j=1}^{n}S_{ij}$, and $\lambda_{2}$ is a trade-off parameter.

To learn the consensus low-dimensional representation *W*, the consensus co-cluster affinity matrix *S* and the cluster indicator matrix *H* from multiple omics data and multiple base clustering results jointly, we combine Equations (4) and (9), and obtain the final objective function of our GRMEC-SC model as follows:

(10) $$\begin{array}{c} \begin{array}{c} \underset{W,V^{v},H,S,w_{v},\alpha_{rv}}{\min}L=L_{MV}\;+\;L_{EC}\\ =\sum_{v}w_{v}||X^{v}-WV^{v}|{|}_{F}^{2}\;+\;\lambda_{1}tr(W^{T}L_{H}W)\;+\;\\ \sum_{r,v}\alpha_{rv}||S-S^{rv}|{|}_{F}^{2}\;+\;\lambda_{2}tr(H^{T}L_{S}H),\\ s.t.W\;>\;0,V^{v}\;>\;0,S\;>\;0,H\;>\;0,H_{i\cdot }1_{c}=1,\\ v=1,\ldots ,m, \end{array} \end{array}$$

where $\lambda_{1}$ and $\lambda_{2}$ are the trade-off parameters. The true cluster index *z* of *i*th cell falls on the maximum value $H_{iz}$ of row vector $H_{i\cdot }$, which means *i*th cell is most likely belong to *z*th cluster. This property allows us to directly obtain the clustering result by iteratively locating the largest index for each row of *H*.

Algorithm 1 Graph Regularized Multi-view Ensemble Clustering Model for Single-Cell Clustering

**Input:** Single-cell multi-omics data $X^{v}$, 1≤v≤m, base clustering results $P^{rv}$, 1≤r≤q, 1≤v≤m, parameters $\lambda_{1}$, $\lambda_{2}$, number of clusters *C*.

**Output:** Cluster indicator matrix *H* and cluster labels corresponding to the maxima of each row of *H*.

1: **Initialize** *W*, $V^{v}$, *S*, and *H* randomly, $w_{v}=\frac{1}{m}$, $\alpha_{rv}=\frac{1}{qm}$;

2: **while**$\left|\frac{L^{t-1}-L^{t}}{L^{t}}\right|>\epsilon$ and t≤MaxIterations**do**

3:  Update *W* with Eq. (12);

4:  Update $V^{v}$ with Eq. (14);

5:  Update *S* with Eq. (16);

6:  Update *H* with Eq. (18) and unitize *H* by row;

7:  Update the weight $w_{v}$ and $\alpha_{rv}$ with Eq. (3) and Eq. (8);

8:  Calculate objective function $L^{t}$ by Eq. (10);

9: **end while**

### 2.2 Optimization

Similar to previous studies (Nie *et al.* 2017a,b), we solve the above problem by using an alternating iterative optimization strategy. In particular, we split the overall objective function into several subproblems and solve them individually with respect to one variable while fixing the others. In the meantime, $w_{v}$ and $\alpha_{rv}$ will be updated according to Equation (3) and (8) respectively.

Solving Equation (10) with respect to *W* is equivalent to:

(11) $$\begin{array}{c} \begin{array}{cc} & \underset{W}{\min}\sum_{v}w_{v}||X^{v}-WV^{v}|{|}_{F}^{2}\;+\;\lambda_{1}tr(W^{T}L_{H}W),\\ & s.t.\;W\;>\;0. \end{array} \end{array}$$

Taking the derivative of Equation (11) with respect to *W* and set it to zero, we can obtain the following updating rule of *W* as:

(12) $$\begin{array}{c} \begin{array}{c} W=W\cdot \frac{\sum_{v}w_{v}X^{v}V^{v^{T}}}{\lambda_{1}L_{H}W\;+\;W(\sum_{v}w_{v}V^{v}V^{v^{T}})}. \end{array} \end{array}$$

Solving Equation (10) with respect to $V^{v}$ is equivalent to:

(13) $$\begin{array}{c} \begin{array}{c} \underset{V^{v}}{\min}w_{v}||X^{v}-WV^{v}|{|}_{F}^{2},\\ s.t.\;V^{v}>0. \end{array} \end{array}$$

The updating rules of $V^{v}$ can be deduced as follows:

(14) $$\begin{array}{c} \begin{array}{c} V_{v}=V^{v}\cdot \frac{W^{T}X^{v}}{W^{T}WV^{v}}. \end{array} \end{array}$$

Solving Equation (10) with respect to *S* is equivalent to:

(15) $$\begin{array}{c} \begin{array}{c} \underset{S}{\min}\sum_{r,v}\alpha_{rv}||S-S^{rv}|{|}_{F}^{2}\;+\;\lambda_{2}tr(H^{T}L_{S}H)\\ =\sum_{r,v}\alpha_{rv}tr(SS^{T}-2SS^{rv^{T}})\;+\;\frac{\lambda_{2}}{2}tr(D^{h}S^{T}),\\ s.t.\;S>0. \end{array} \end{array}$$

where $D_{ij}^{h}=||H_{i\cdot }-H_{j\cdot }|{|}_{2}^{2}$. The updating rule of *S* is as follows:

(16) $$\begin{array}{c} \begin{array}{c} S=S\cdot \frac{4\sum_{rv}\alpha_{rv}S^{rv}}{4\sum_{rv}\alpha_{rv}S\;+\;\lambda_{2}D^{h}}. \end{array} \end{array}$$

Solving Equation (10) with respect to *H* is equivalent to:

(17) $$\begin{array}{c} \begin{array}{c} \underset{H}{\min}\lambda_{1}tr(W^{T}L_{H}W)\;+\;\lambda_{2}tr(H^{T}L_{S}H),\\ s.t.\;H\;>\;0,H_{i.}1_{c}=1. \end{array} \end{array}$$

where $D_{ij}^{W}=||W_{i\cdot }-W_{j\cdot }|{|}_{2}^{2}$. The updating rule of *H* is as follows:

(18) $$\begin{array}{c} \begin{array}{c} H=H\cdot \frac{\lambda_{2}SH}{(\frac{\lambda_{1}}{2}D^{W}+\lambda_{2}D^{S})H}. \end{array} \end{array}$$

### 2.3 Complexity analysis

The time complexity of updating *W*, $V^{v}$, *S* and *H* once are $O((\sum_{v=1}^{m}p_{v})(n\;+\;k)k\;+\;n^{2}k)$, $O(p_{v}nk\;+\;nk^{2}\;+\;k^{2}p_{v})$, $O(n^{2})$ and $O(n^{2}C)$ respectively (*k* is typically smaller than *n*). Therefore, the total time complexity of updating our model once is $O((\sum_{v=1}^{m}p_{v})nk\;+\;n^{2}(k\;+\;C)\;+\;k^{2}p_{v})$.

## 3 Experiments

In this section, we thoroughly evaluate the performance of our GRMEC-SC method on five single-cell multi-omics datasets. We extensively compare GRMEC-SC with both mono-omic clustering and multi-omics clustering algorithms.

### 3.1 Experimental setting

#### 3.1.1 Datasets

To evaluate the performance of various models on different types of single-cell multi-omics data, we collect five different single-cell multi-omics datasets, including Inhouse (Wang *et al.* 2020), Public (Wang *et al.* 2020), 10x-pbmc-3k (https://www.10xgenomics.com/resources/datasets/pbmc-from-a-healthy-donor-no-cell-sorting-3-k-1-standard-2-0-0), Ma-2020 (Ma *et al.* 2020), and cellMix (Chen *et al.* 2019). Here, Inhouse and Public are both CITE-seq data which measure matched transcriptome and surface protein data, while 10x-pbmc-3k, Ma-2020 and cellMix include matched scRNA-seq and scATAC-seq data.

We use the preprocessing strategy utilized in Seurat (Hao *et al.* 2021) to preprocess scRNA-seq data and select the top 1000 highly variable genes (HVGs) for each scRNA-seq dataset. For ATAC-seq data, we utilize the Signac (Stuart *et al.* 2021) package to select peaks. Furthermore, for each dataset, we calculate the number of genes with zero expression for each cell and eliminate cells with a high number of genes having expression values of 0. Table 1 summarizes the details of the real datasets used in this study.

**Table 1.** Statistics of the single-cell multi-omics datasets.

Dataset	Cells	Genes	Peaks	Proteins	Cell types
Public	3528	33 538		14	8
Inhouse	1182	33 538		10	6
cellMix	1047	18 666	136 771		4
10x-pbmc-3k	2883	21 342	72 540		8
Ma-2020	3339	21 478	340 341		22

#### 3.1.2 Evaluation metrics

Two evaluation criteria, i.e. Adjusted rand index (ARI) (Hubert and Arabie 1985) and Normalized Mutual Information (NMI) (McDaid *et al.* 2011), are used for assessing the performance of various methods. For detailed definition of these two metrics, please refer to Supplementary Materials.

#### 3.1.3 Benchmark and parameter settings

We compare the performance of GRMEC-SC with five mono-omic clustering algorithms and eight multi-omics clustering methods on five single-cell multi-omics datasets. Specifically, we compare our model with five popular scRNA-seq data clustering algorithms, i.e. CIDR (Lin *et al.* 2017), CountClust (Dey *et al.* 2017), SC3 (Kiselev *et al.* 2017), Seurat (Stuart *et al.* 2019), and SIMLR (Wang *et al.* 2017). Note that we also use these five methods as base clustering methods to generate base clustering results for each dataset. As these mono-omic clustering methods cannot handle multi-omics data, we apply them on each omic data separately and use their clustering results as the base clustering results for our model. Given that existing multi-omics clustering models are typically tailored for a particular type of multi-omics data, we specifically include a comparison with BREM-SC (Wang *et al.* 2020), CITEMO (Hu *et al.* 2022), and CiteFuse (Kim *et al.* 2020) on the CITE-seq datasets, which are designed for CITE-seq data. For multi-omics datasets containing both scRNA-seq and scATAC-seq data, we include the comparison with JSNMF (Ma *et al.* 2022), scMCs (Ren *et al.* 2023), and MOFA+ (Argelaguet *et al.* 2020). In addition, we also compare our model with five popular multi-view clustering methods, including MVEC (Tao *et al.* 2017), SWMC (Nie *et al.* 2017b), MVAN (Nie *et al.* 2017a), CSMV (Luo *et al.* 2018), and Seurat-WNN (Hao *et al.* 2021).

To ensure a fair comparison, the number of clusters for all clustering methods is set to the true number of cell clusters, except for CIDR and Seurat, which have the capability to adaptively predict the number of clusters. For our GRMEC-SC model, we select the optimal hyper-parameters $\lambda_{1}$ and $\lambda_{2}$ from range $\left\{10^{-4},10^{-3},\ldots ,10^{4}\right\}$. The same parameter tuning process is carried out for the other comparison methods to achieve their optimal results, with the exception of SWMC, MVAN, and JSNMF. SWMC is a parameter-free algorithm, while MVAN and JSNMF automatically adjust the trade-off parameter depending on the number of connected components in the consensus matrix.

## 4 Results

### 4.1 Parameter sensitivity

There are two trade-off hyper-parameters $\lambda_{1}$ and $\lambda_{2}$ in our GRMEC-SC model. $\lambda_{1}$ controls the agreement between the low-dimensional representation *W* and the cluster indicator matrix *H*, while $\lambda_{2}$ controls the effect of the consensus co-cluster affinity matrix *S* on the final clustering results. To assess the sensitivity of GRMEC-SC to these parameters, a parameter sensitivity experiment is conducted. The performance of GRMEC-SC is assessed using NMI and ARI metrics. The values of $\lambda_{1}$ and $\lambda_{2}$ are selected from $\left\{10^{-4},10^{-3},\ldots ,10^{4}\right\}$.

**Figure 2.**
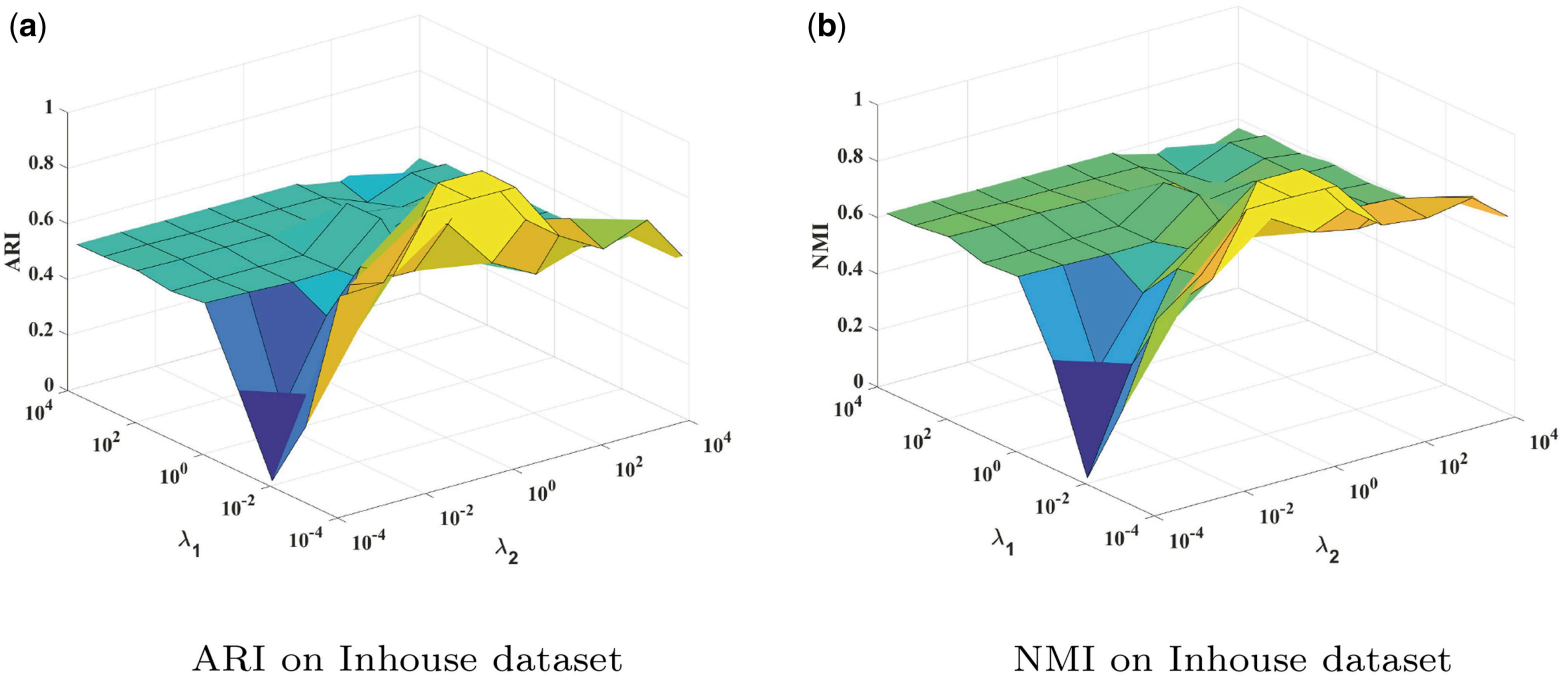
Sensitivity analysis of the hyper-parameters on the Inhouse dataset. (a) ARI on Inhouse dataset. (b) NMI on Inhouse dataset.

The performance of GRMEC-SC with varying values of $\lambda_{1}$ and $\lambda_{2}$ on the Inhouse dataset is depicted in Fig. 2. Results for the remaining datasets can be found in Supplementary Figs S2–S5. From these figures, it is evident that when $\lambda_{1}$ and $\lambda_{2}$ are sufficiently large, the performance of GRMEC-SC remains relatively stable. Based on these results, we recommend setting the default values of $\lambda_{1}$ and $\lambda_{2}$ to be 0.1 and 0.01.

### 4.2 Clustering results

**Figure 3.**
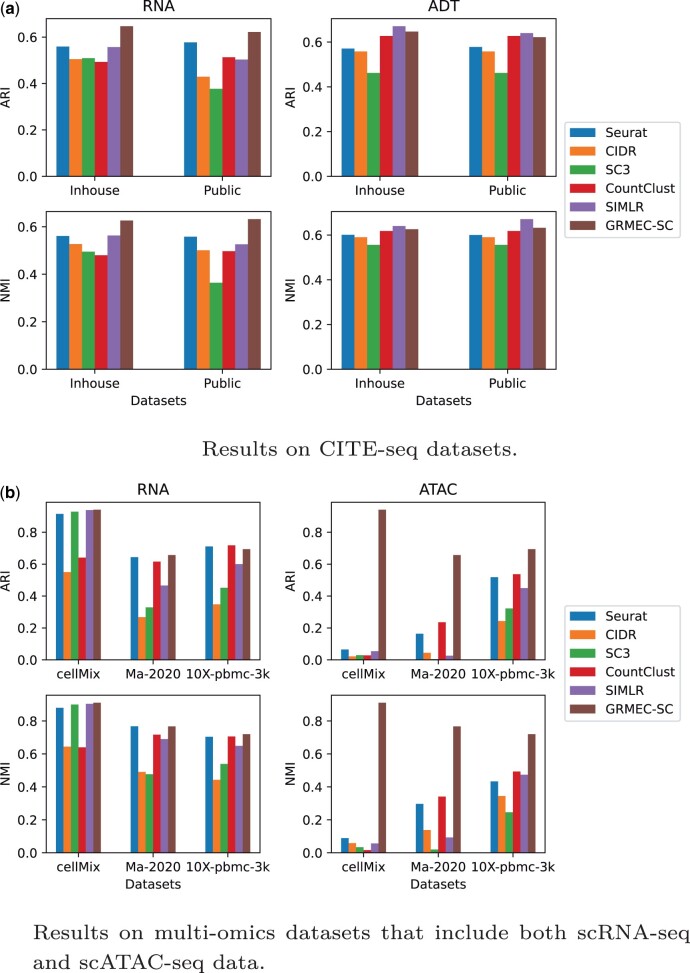
The clustering performance of GRMEC-SC and various base clustering methods is evaluated on different datasets. Each base clustering method is applied individually to each omic data to assess its performance. (a) Results on CITE-seq datasets. (b) Results on multi-omics datasets that include both scRNA-seq and scATAC-seq data.

Firstly, we compare GRMEC-SC with five mono-omic clustering methods, which are also utilized as base clustering methods. The results are depicted in Fig. 3. It can be observed from Fig. 3a that on the two CITE-seq datasets, our GRMEC-SC outperforms these base clustering methods in most cases in terms of the two evaluation metrics. From Fig. 3b, we can see that on the other three multi-omics datasets, GRMEC-SC still demonstrates competitive performance compared to the base clustering methods. In addition, the performance of each base clustering method varies across different datasets, with none of them dominating the others consistently. Since the base clustering methods are designed primarily for clustering scRNA-seq data and the characteristics of scATAC-seq data differ from those of scRNA-seq data, these methods show suboptimal performance on the scATAC-seq data from the cellMix and Ma-2020 datasets. By introducing a weighted ensemble learning strategy to integrate multiple base clustering results and incorporating the original omics data to reduce the model’s dependence on base clustering results, our method achieves stable and robust performance across all cases.

Furthermore, we compare GRMEC-SC with eleven multi-omics clustering methods, and the corresponding results are shown in Tables 2 and 3. From these tables, it is evident that our GRMEC-SC can outperform other compared methods in the majority of cases, highlighting the effectiveness of our representation learning and ensemble learning strategies. Unlike existing multi-omics clustering methods that are tailored for specific data types and may not be applicable to other data types, our GRMEC-SC is versatile in handling various types of multi-omics data and consistently achieves strong performance.

**Table 2.** The performance of various methods on the multi-omics datasets which include both scRNA-seq and scATAC-seq data.^a^

Datasets	Evaluation metrics	Methods								
		MVEC	MVAN	SWMC	CSMV	Seurat-WNN	JSNMF	scMCs	MOFA+	GRMEC-SC
cellMix	ARI	0.682	0.007	0.164	0.929	0.935	0.385	0.888	0.154	**0.941**
	NMI	0.675	0.026	0.341	0.903	0.902	0.474	0.892	0.272	**0.911**
Ma-2020	ARI	0.542	0.009	0.007	0.626	**0.690**	0.630	0.388	0.049	0.657
	NMI	0.684	0.055	0.256	0.762	**0.842**	0.745	0.599	0.191	0.767
10X-pbmc-3k	ARI	0.369	0.040	0.467	0.637	0.389	0.632	0.435	0.295	**0.694**
	NMI	0.592	0.017	0.588	0.655	0.539	**0.723**	0.639	0.322	0.720

a Optimal values are highlighted in bold and second best values are underlined.

**Table 3.** The performance of various methods on the CITE-seq datasets.^a^

Datasets	Evaluation metrics	Methods								
		MVEC	MVAN	SWMC	CSMV	Seurat-WNN	BREM-SC	CITEMO	CiteFuse	GRMEC-SC
Inhouse	ARI	0.767	0.133	0.650	0.981	0.337	0.885	0.338	0.953	**0.996**
	NMI	0.891	0.269	0.804	0.946	0.542	0.886	0.678	0.912	**0.979**
Public	ARI	0.388	0.406	0.463	0.506	**0.661**	0.477	0.650	0.648	0.647
	NMI	0.504	0.455	0.505	0.503	0.608	0.511	**0.626**	**0.626**	**0.626**

a Optimal values are highlighted in bold and second best values are underlined.

Our GRMEC-SC has the capability to learn consensus low-dimensional representations of cells from multi-omics data. To demonstrate the effectiveness of our model in learning cell representations, we compare the UMAP visualization of the original scRNA-seq data with our learned representations. Supplementary Fig. S6 illustrates the UMAP visualization on two CITE-seq datasets. Supplementary Fig. S6a and c displays the results obtained from the raw scRNA-seq data, while Supplementary Fig. S6b and d presents the results derived from the consensus low-dimensional representation *W* learned by our GRMEC-SC. It is apparent from these figures that distinguishing different cell types from raw scRNA-seq data is challenging, whereas our learned low-dimensional representation facilitates a clearer differentiation of various cell types.

### 4.3 Marker gene identification

**Figure 4.**
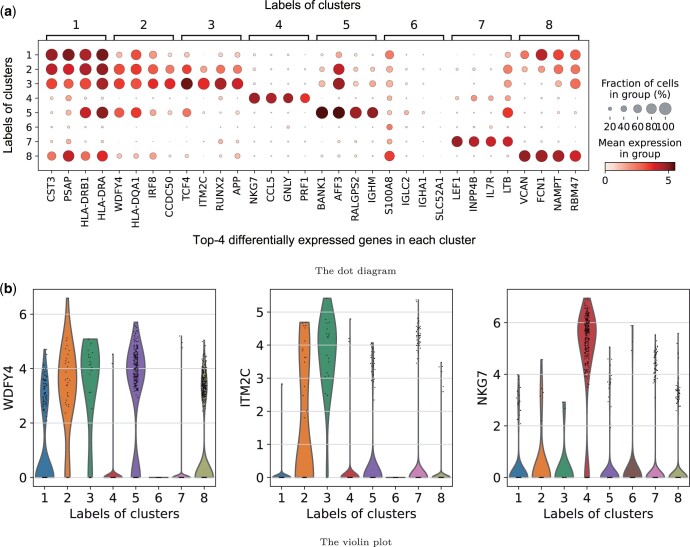
Our identified marker genes on the 10X-pbmc-3k dataset. (a) Dot diagram illustrating the average expression of the top-4 differentially expressed genes within each predicted cluster. (b) Violin plots showcasing the identified marker genes across different clusters.

Marker genes are highly expressed genes in specific cell types. It is worth noting that finding the marker genes of cell clusters is an important step toward cell type annotation. Here, we use Wilcoxon rank-sum test to detect differential expression genes for each identified cluster and ranked them based on *P*-values. The identified top-4 differentially expressed genes of each cluster detected from the 10X-pbmc-3k dataset are shown in Fig. 4. We can find from Fig. 4a that the marker genes of all clusters except the first three are distinct. This observation may be attributed to the similarity among these three cell types, making it challenging to differentiate them directly based on marker genes alone. Integrating gene expression levels and gene distribution could potentially enhance the ability to distinguish these cell types more effectively. Figure 4b shows the expression levels of three marker genes in different cell clusters. It is clear that the expression level of the WDFY4 is higher in cluster 2 than in other clusters, and the expression levels of the ITM2C in cluster 3 and the NKG7 in cluster 4 are significantly higher than in other clusters, indicating their biological interpretability. These visualization results are obtained using the Scanpy package (Wolf *et al.* 2018).

## 5 Conclusion and discussion

In this study, a novel graph-regularized multi-view clustering model, named GRMEC-SC, has been developed for clustering single-cell multi-omics data. Unlike previous studies that primarily focus on clustering mono-omic data or specific types of multi-omics data, our model is designed to handle various types of multi-omics data with diverse characteristics. Specifically, our model can simultaneously learn the consensus low-dimensional representations and the consensus co-cluster affinity matrix of cells from multiple omics data and multiple base clustering results. By leveraging information from different data sources, our model demonstrates effectiveness across different types of multi-omics data. The performance of our model is rigorously evaluated through comprehensive experiments on five diverse multi-omics datasets. The experimental results showcase the superiority of our model compared to other state-of-the-art clustering methods.

It is essential to recognize that the computational complexity of our model presents challenges, especially when handling large-scale datasets. With the continuous advancements in sequencing technologies resulting in the generation of vast amounts of single-cell data, addressing the computational hurdles linked to such extensive datasets will be a key area of focus in our future research pursuits. We aim to explore strategies for efficient clustering on large-scale datasets, thereby improving the applicability and scalability of our model.

## Supplementary Material

btae169_Supplementary_Data

## Data Availability

All datasets utilized in this study are publicly available, and the source code is accessible online at https://github.com/polarisChen/GRMEC-SC.
