# Supplementary material for: Clustering single-cell multi-omics data via graph regularized multi-view ensemble learning
Source: Bioinformatics. 2024 Mar 28;40(4):btae169. doi: 10.1093/bioinformatics/btae169 (PMC11015955; doi:10.1093/bioinformatics/btae169)
Supplement: btae169_Supplementary_Data [file btae169_supplementary_data.pdf]

Clustering single-cell multi-omics data via graph regularized multi-view ensemble learning

Fuqun Chen, Guanhua Zou, Yongxian Wu, Le Ou-Yang

1 Evaluation Metrics

Two evaluation criteria, i.e., Adjusted rand index (ARI) [1] and Normalized Mutual Information (NMI) [2], are used for assessing the performance of various methods. Given the true cluster labels $\mathbb{C}^* = \{\mathbb{C}^*_a | a = 1, 2, ..., k^a\}$ and the predicted cluster labels $\mathbb{C}^o = \{\mathbb{C}^o_b | b = 1, 2, ..., k^b\}$, where k^a and k^b denote the number of true and predicted clusters respectively. Let $\mathbb{C}^*_a$ be the a-th true cluster which contains n^*_a objects, $\mathbb{C}^o_b$ be the b-th predicted cluster which contains n^o_b objects.

The overlap between two sets of cluster labels $\mathbb{C}^*_a$ and $\mathbb{C}^o_b$ can be summarized as a $k^a \times k^b$ matrix $M = [n_{ab}]^{a=1,...,k^a}_{b=1,...,k^b}$, where $n_{ab} = |\mathbb{C}^*_a \cap \mathbb{C}^o_b|$ denotes the number of objects that belong to both clusters $\mathbb{C}^*_a$ and $\mathbb{C}^o_b$. Moreover, $n = \sum_{a=1}^{k^a} n^*_a = \sum_{b=1}^{k^b} n^o_b$ is the total number of objects.

The entropy associated with true cluster labels $\mathbb{C}^*$ and predicted cluster labels $\mathbb{C}^o$ are $H(\mathbb{C}^*) = -\sum_{a=1}^{k^a} \frac{n^*_a}{n} log(\frac{n^*_a}{n})$ and $H(\mathbb{C}^o) = -\sum_{b=1}^{k^b} \frac{n^o_b}{n} log(\frac{n^o_b}{n})$, respectively. The joint entropy of $\mathbb{C}^*$ and $\mathbb{C}^o$ is $H(\mathbb{C}^*, \mathbb{C}^o) = -\sum_{a=1}^{k^a} \sum_{b=1}^{k^b} \frac{n_{ab}}{n} log(\frac{n_{ab}}{n})$. Thus, the NMI between $\mathbb{C}^*$ and $\mathbb{C}^o$ is defined as:

$$\text{NMI} = \frac{H(\mathbb{C}^*) + H(\mathbb{C}^o) - H(\mathbb{C}^*, \mathbb{C}^o)}{\sqrt{H(\mathbb{C}^*)H(\mathbb{C}^o)}} = \frac{I(\mathbb{C}^*, \mathbb{C}^o)}{\sqrt{H(\mathbb{C}^*)H(\mathbb{C}^o)}}. \quad (1)$$

where $I(\mathbb{C}^*, \mathbb{C}^o)$ is the mutual information between two sets of cluster labels $\mathbb{C}^*$ and $\mathbb{C}^o$.

ARI measures the similarity between true cluster labels $\mathbb{C}^*$ and predicted cluster labels $\mathbb{C}^o$, which is defined as:

$$\text{ARI} = \frac{\sum_{a,b}\binom{n_{ab}}{2} - [\sum_{a}\binom{n^*_a}{2}\sum_{b}\binom{n^o_b}{2}]/\binom{n}{2}}{\frac{1}{2}[\sum_{a}\binom{n^*_a}{2} + \sum_{b}\binom{n^o_b}{2}] - [\sum_{a}\binom{n^*_a}{2}\sum_{b}\binom{n^o_b}{2}]/\binom{n}{2}}. \quad (2)$$

where $\binom{a}{b}$ is the combination formula.

2 Supplementary Figure

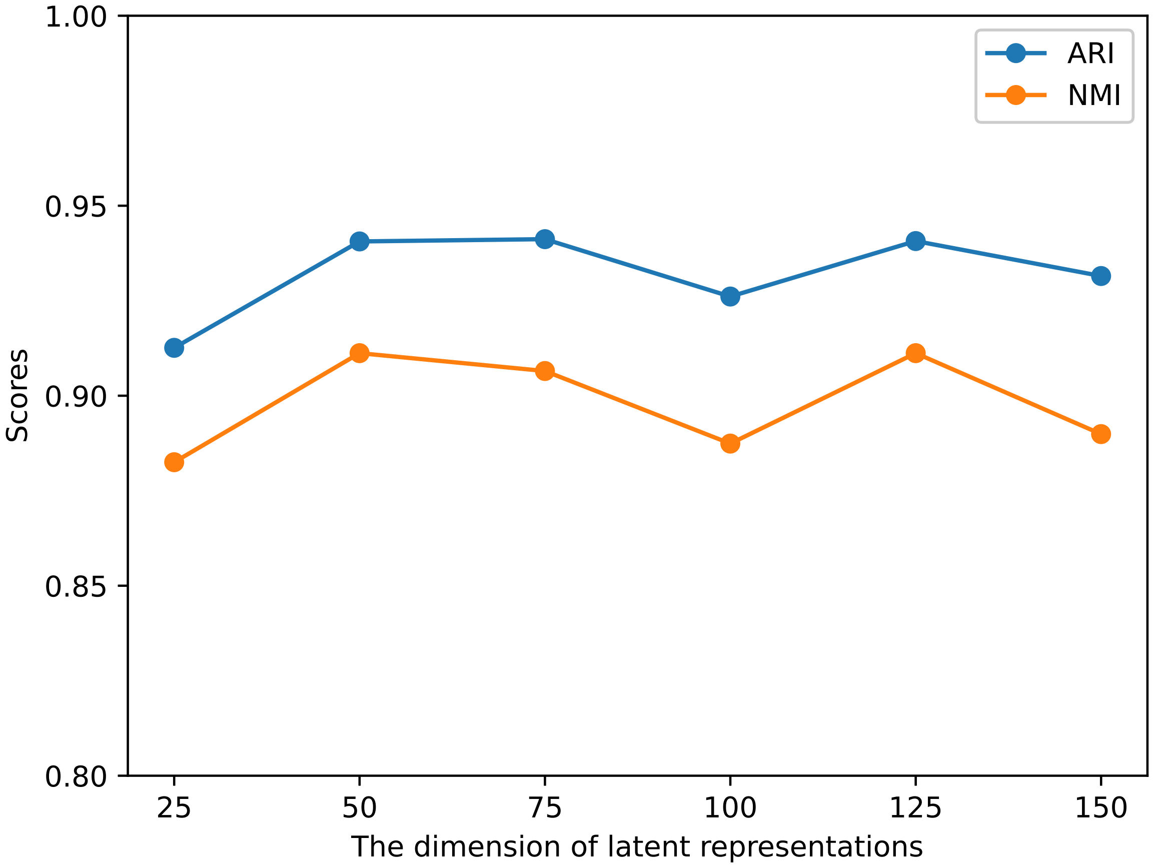

Figure S1: The impact of the latent representation dimension k on the cellMix dataset was assessed by varying k within the range value of $\{25, 50, \ldots, 150\}$. Evaluation of the Adjusted Rand Index (ARI) and Normalized Mutual Information (NMI) values across different latent representation dimensions revealed that the clustering performance is optimal at latent representation dimensions of 50 and 125. Therefore, we have chosen 50 as the default value of k in our study.

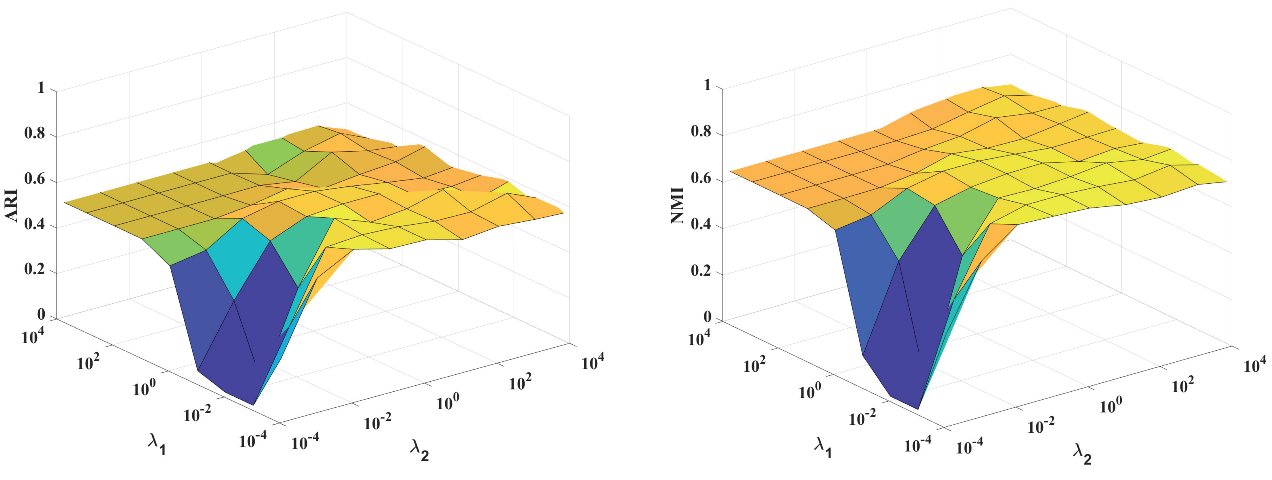

(a) ARI on Ma-2020 dataset

(b) NMI on Ma-2020 dataset

Figure S2: Sensitivity analysis of the hyper-parameters on the Ma-2020 dataset.

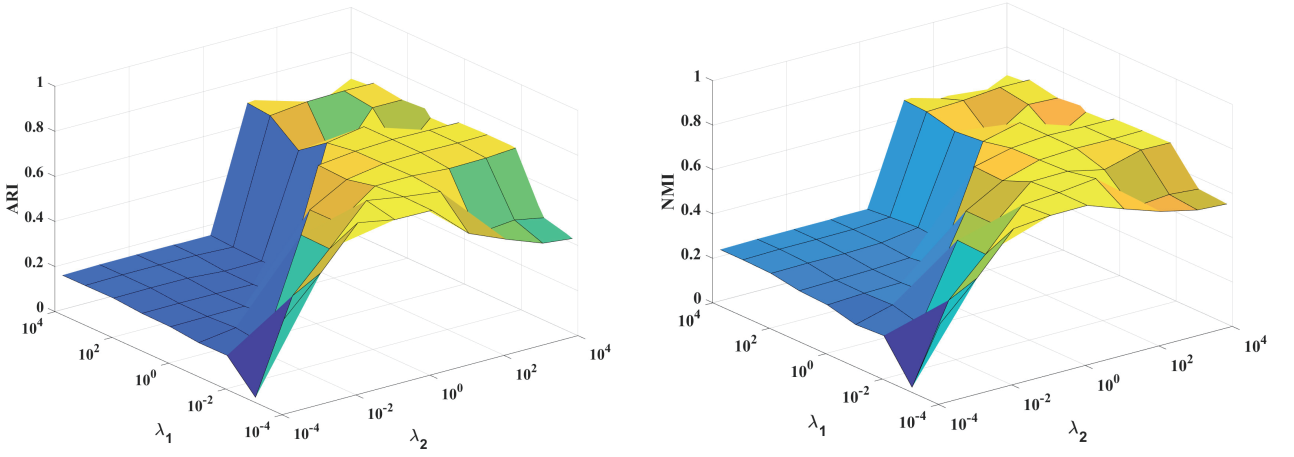

(a) ARI on 10x-pbmc-3k dataset (b) NMI on 10x-pbmc-3k dataset

Figure S3: Sensitivity analysis of the hyper-parameters on the 10x-pbmc-3k dataset.

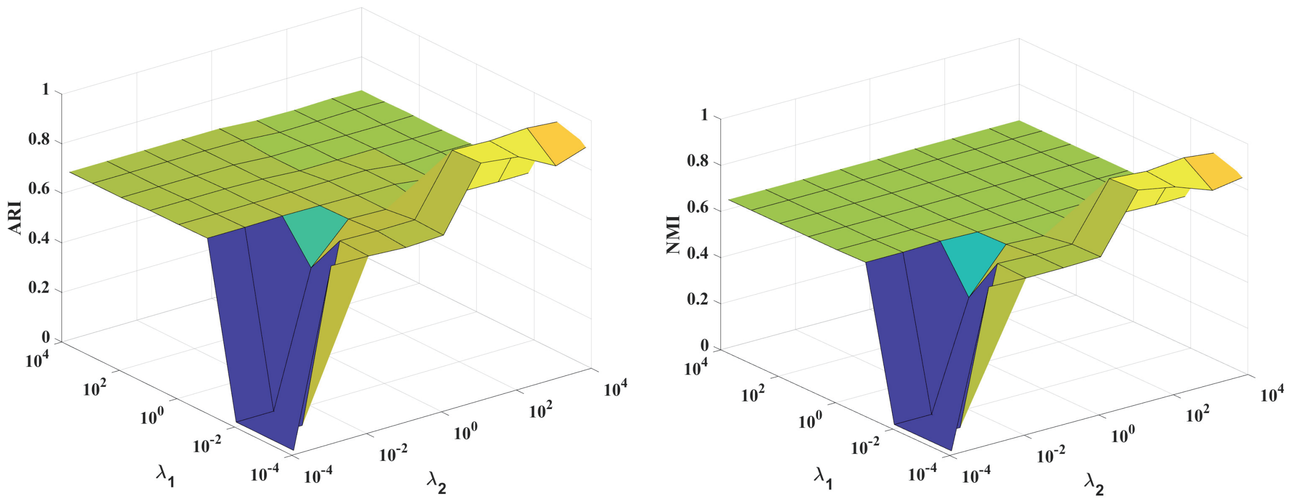

(a) ARI on cellMix dataset (b) NMI on cellMix dataset

Figure S4: Sensitivity analysis of the hyper-parameters on the cellMix dataset.

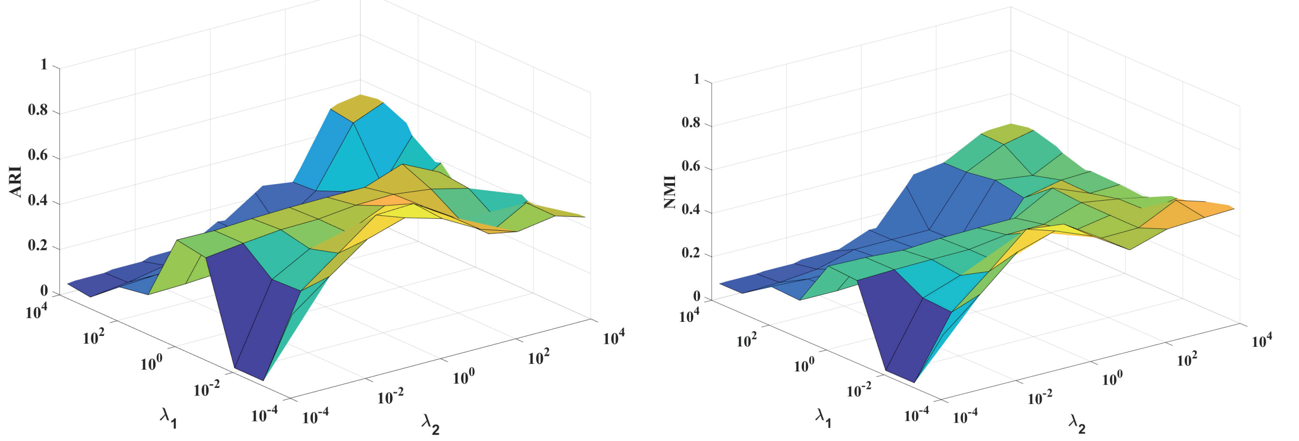

(a) ARI on Public dataset (b) NMI on Public dataset

Figure S5: Sensitivity analysis of the hyper-parameters on the Public dataset.

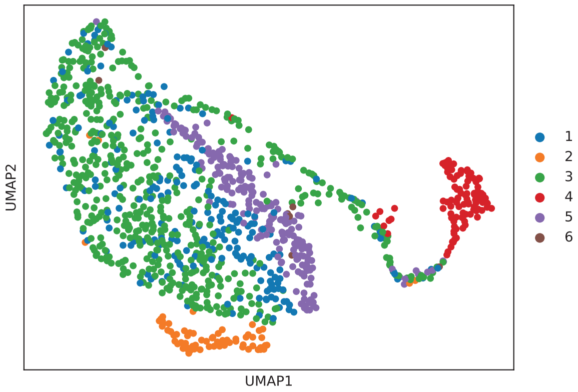

(a) UMAP of the raw data on Inhouse dataset

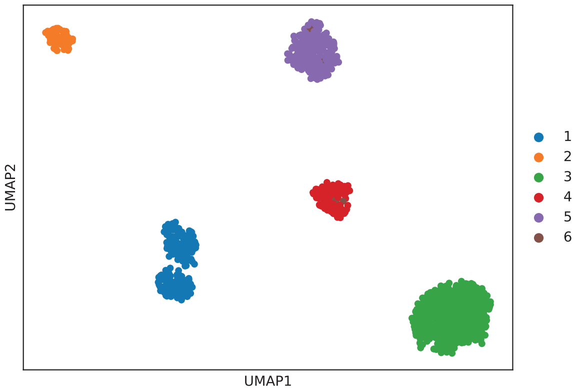

(b) UMAP of the low-dimensional representation on Inhouse dataset

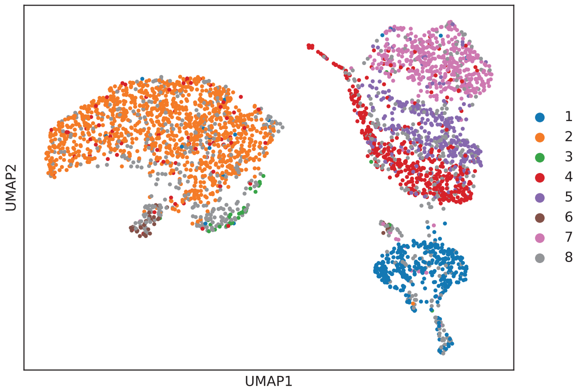

(c) UMAP of the raw data on Public dataset

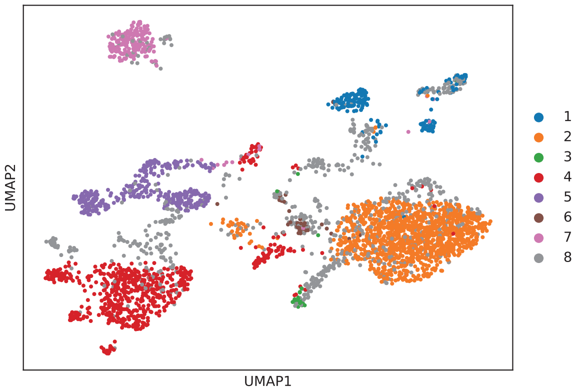

(d) UMAP of the low-dimensional representation on Public dataset

Figure S6: UMAP visualization of cells on the Inhouse and Public datasets. Left: UMAP visualization of raw data; Right: UMAP visualization of the consensus low-dimensional representations learned by our GRMEC-SC.

References

[1] Lawrence Hubert and Phipps Arabie. Comparing partitions. Journal of classification, 2:193–218, 1985.

[2] Aaron F McDaid, Derek Greene, and Neil Hurley. Normalized mutual information to evaluate overlapping community finding algorithms. arXiv preprint arXiv:1110.2515, 2011.
